# Dealing with dimensionality: the application of machine learning to multi-omics data

**DOI:** 10.1093/bioinformatics/btad021

**Published:** 2023-01-13

**Authors:** Dylan Feldner-Busztin, Panos Firbas Nisantzis, Shelley Jane Edmunds, Gergely Boza, Fernando Racimo, Shyam Gopalakrishnan, Morten Tønsberg Limborg, Leo Lahti, Gonzalo G de Polavieja

**Affiliations:** Champalimaud Centre for the Unknown, Champalimaud Foundation, 1400-038 Lisbon, Portugal; Champalimaud Centre for the Unknown, Champalimaud Foundation, 1400-038 Lisbon, Portugal; Center for Evolutionary Hologenomics, GLOBE Institute, Faculty of Health and Medical Sciences, University of Copenhagen, 1353 Copenhagen, Denmark; Centre for Ecological Research, 1113 Budapest, Hungary; Faculty of Health and Medical Sciences, University of Copenhagen, 2200 Copenhagen, Denmark; Center for Evolutionary Hologenomics, GLOBE Institute, Faculty of Health and Medical Sciences, University of Copenhagen, 1353 Copenhagen, Denmark; Center for Evolutionary Hologenomics, GLOBE Institute, Faculty of Health and Medical Sciences, University of Copenhagen, 1353 Copenhagen, Denmark; Department of Computing, University of Turku, 20014 Turku, Finland; Champalimaud Centre for the Unknown, Champalimaud Foundation, 1400-038 Lisbon, Portugal

## Abstract

**Motivation:**

Machine learning (ML) methods are motivated by the need to automate information extraction from large datasets in order to support human users in data-driven tasks. This is an attractive approach for integrative joint analysis of vast amounts of omics data produced in next generation sequencing and other -omics assays. A systematic assessment of the current literature can help to identify key trends and potential gaps in methodology and applications. We surveyed the literature on ML multi-omic data integration and quantitatively explored the goals, techniques and data involved in this field. We were particularly interested in examining how researchers use ML to deal with the volume and complexity of these datasets.

**Results:**

Our main finding is that the methods used are those that address the challenges of datasets with few samples and many features. Dimensionality reduction methods are used to reduce the feature count alongside models that can also appropriately handle relatively few samples. Popular techniques include autoencoders, random forests and support vector machines. We also found that the field is heavily influenced by the use of The Cancer Genome Atlas dataset, which is accessible and contains many diverse experiments.

**Availability and implementation:**

All data and processing scripts are available at this GitLab repository: https://gitlab.com/polavieja_lab/ml_multi-omics_review/ or in Zenodo: https://doi.org/10.5281/zenodo.7361807.

**Supplementary information:**

Supplementary data are available at *Bioinformatics* online.

## 1 Introduction

Algorithmic and hardware developments, including graphics processing unit computation, have spurred a revolution in machine learning (ML) (Dally *et al.*, 2021). At the same time, the amount of data generated by -omic (genomic, transcriptomic, etc.) high-throughput-sequencing and other techniques has been growing exponentially (Lightbody *et al.*, 2019). This growing body of information needs statistical models that can extract accurate and explainable predictions from it.

The concept of ‘training’ defines ML techniques. A subset of the original data is used to train, or change the model’s parameters, so that the model can then make the best possible predictions or decisions. ML models typically work better with large training datasets and as such should be particularly well tailored for multi-omic data integration. The ML field is an exciting frontier and several reviews have been published in this area recently. Reel *et al.* (2021) rated ML algorithms based on their data-hungriness, prediction accuracy and other characteristics. The review by Cai *et al.* (2022) highlighted the use of The Cancer Genome Atlas (TCGA) in multi-omics research, including further independent benchmarking of ML techniques on another dataset—the Cancer Cell Line Encyclopedia (Ghandi *et al.*, 2019; Nusinow *et al.*, 2020). Marcos-Zambrano (2021) and Moreno-Indias (2021) explored ML multi-omics in the context of microbiome research. While these reviews have illustrated that ML applications can handle and thrive on large volumes of data from multi-omics datasets there is one caveat: the modelling of these datasets often suffers from the low sample size compared to the vast dimensionality.

Ideally, ML techniques would use more samples than features (Bellman, 1961). In practice, however, a single -omic dataset can contain tens of thousands of features (e.g. the result of RNAseq on a tumour sample can include measurements for over 20 000 human genes). This makes multi-omics datasets high-dimensional. On the other hand, most datasets contain at most only a few hundred samples, i.e. one per subject.

Aware of this dimensionality issue, we set out to explore how prevalent it is in the field of ML data integration in multi-omics, what strategies are used to overcome it, and what can be achieved with ML in multi-omics. We took a quantitative approach to gathering characteristics of papers (e.g. techniques used, goals for using ML) from papers and used the PRISMA-ScR framework (Tricco *et al.*, 2018) as a guideline for performing a more structured review.

Following the PRISMA-ScR guidelines, we developed an explicit statement of the questions being addressed in this scoping review (Table 1).

## 2 Materials and methods

### 2.1 Protocol and registration

**Fig. 1.**
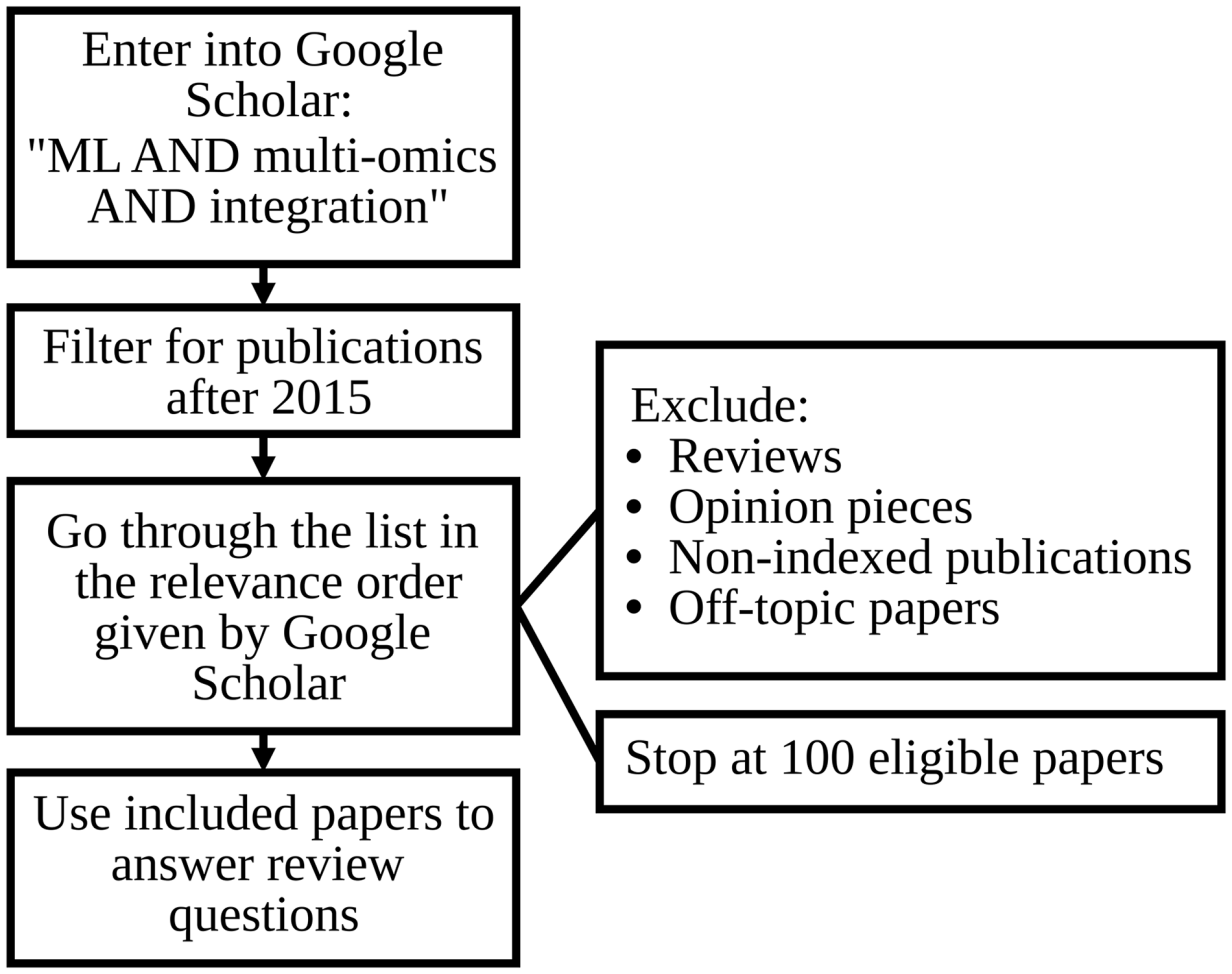
Overview of paper selection process

For this work, we used the PRISMA Extension for Scoping Reviews (PRISMA-ScR) (Tricco, 2018) which is available on the PRISMA website (https://web.archive.org/web/20220322230828/http://www.prisma-statement.org/Extensions/ScopingReviews). A brief overview of our selection process can be found in Figure 1. Further details around the methodology in line with PRISMA-ScR are included in the Supplementary Material.

**Table 1.** Review questions

Review questions	
1.	What is multi-omic data in practice?
1.1.	Which types of -omics features made up the ‘data dimensions’?
1.2.	How is the data structured: how many features versus samples?
2.	What analysis has been done on multi-omics data?
2.1.	Which ML techniques were used?
2.2.	What were the goals of the ML application?
2.3	What were the targets/labels of classification tasks?
3.	Can we explain trends using an analysis of the citation of papers since 2015?

One hundred papers were selected for the main inquiries. Following the same process, we conducted a second search using only the terms: ‘ML AND integration’. For this dataset, many off-topic papers were excluded, as well as any multi-omic papers that were included in the first dataset. This second dataset was used to explore what kind of ML techniques was used in a broader ‘ML integration’ field in comparison to the ‘multi-omic ML integration’ field.

## 3 Results

### 3.1 What is multi-omic data in practice?

#### 3.1.1 Which types of -omics features made up the ‘data dimensions’?

**Fig. 2.**
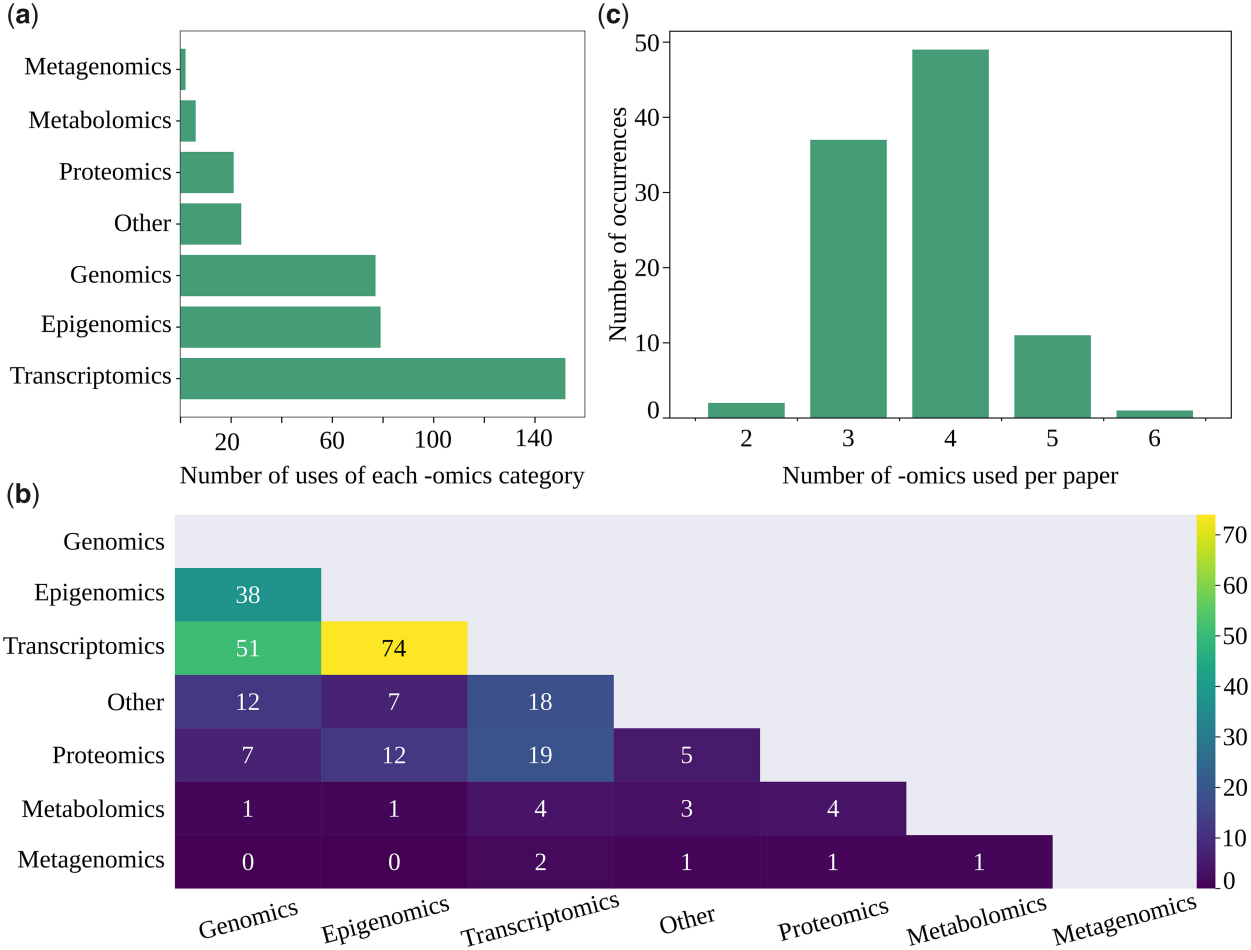
(**a**) Number of uses of each -omics category in the reviewed papers. (**b**) Number of -omics used per paper in the reviewed papers. (**c**) The number of appearances -omics pairs across papers

Using the papers from the ‘ML AND multi-omics AND integration’ search, we compiled the types of -omics data that were used by researchers. Transcriptomics were by far the most popular measurement and were used 152 times (many works use multiple types of transcriptomics such as mRNA and miRNA). This accounted for 42% of all the -omics data uses (Fig. 2a). Epigenomics and genomics data follow with 79 (22%) and 77 uses (21%) respectively. After that were proteomics (21 uses, 6%), metabolomics (6 uses, 2%), metagenomics (2 uses, 1%) and *other* (24 uses, 7%). This distribution is heavily influenced by the TCGA (Tomczak, 2015) database, but the trend remains when excluding this database **(**see Supplementary Fig. S1).

Most papers we surveyed used three or more different -omics types (Fig. 2c). The -omics types that most often appeared together were transcriptomics and epigenomics, followed by transcriptomics and genomics. The top two combinations remained unchanged when we ignored the papers that relied on TCGA (see Supplementary Fig. S1).

#### 3.1.2 How is the data structured: how many features versus samples?

**Fig. 3.**
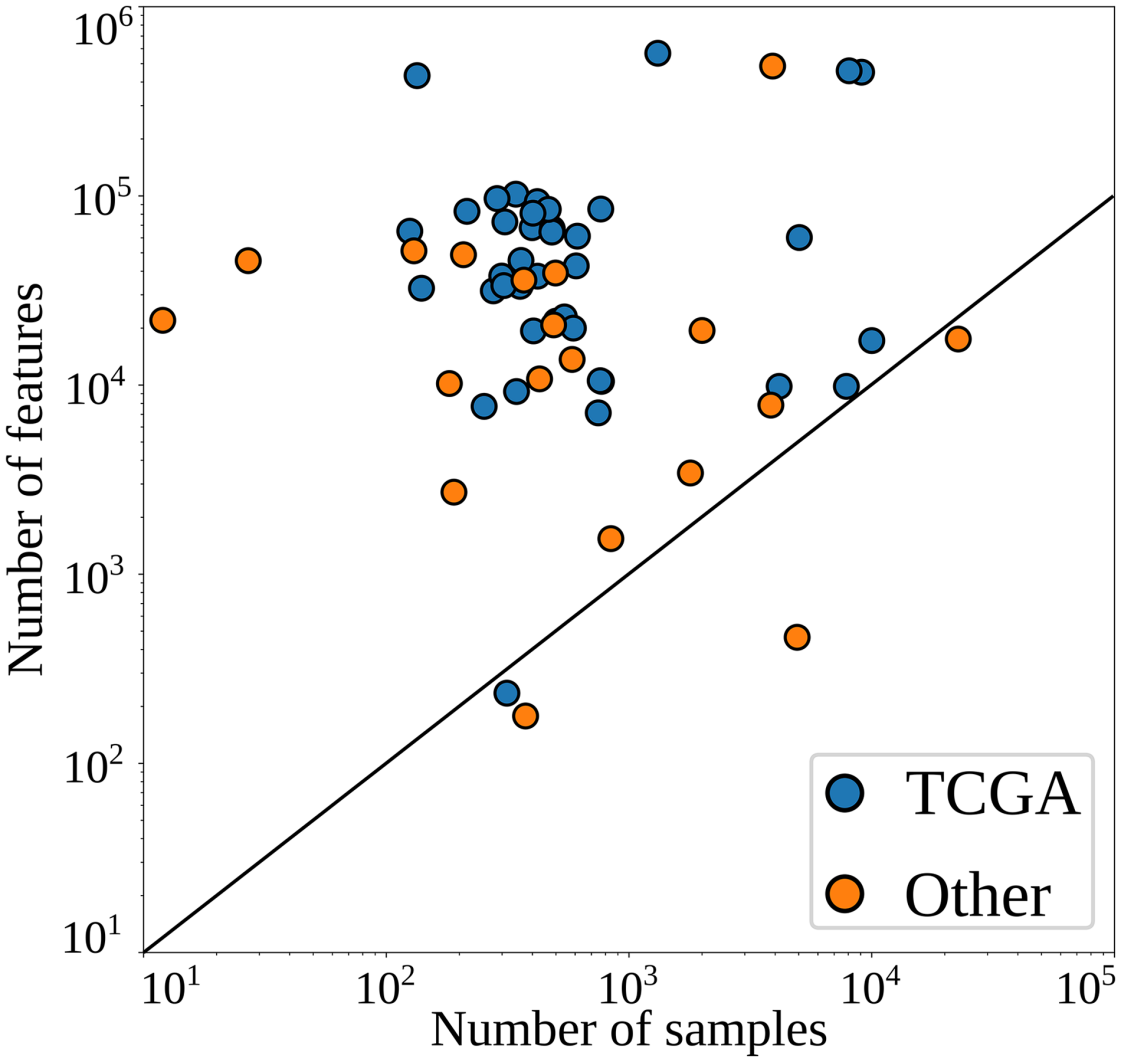
‘Shape’ of multi-omics datasets. Number of samples (*x*-axis), number of features (*y*-axis)

There were far more features than samples in most cases (Fig. 3). The median number of features used in the surveyed publications was 33 415 while the median number of samples was 447. Due to outliers, these are different from the mean number of features, 73 996 and the mean number of samples, 1767. Most multi-omics ML method development research relies on existing data. TCGA was used in 73% of the surveyed papers. Creating multi-omics datasets has high costs in terms of money and time, but also requires a broad range of expertise not often found in a single research group. Besides this, the recent push for FAIR (Findability, Accessibility, Interoperability and Reusability) principles (Wilkinson, 2016) and the open science movement is helping to make data more easily accessible. Now researchers can tap into the community’s shared resources in order to supplement their own data, or simply to experiment with new iterations of ML techniques. Databases where biological data are uniformly processed make this process even easier and allow many different techniques to be tested, facilitating progress.

After splitting publications into those that relied on TCGA and those that relied on *other* datasets, we found no significant difference in the number of samples used, but we did observe a difference in the number of features (*P* < 0.01 using the Mann-Whitney *U* test).

### 3.2 What analysis has been done on multi-omics data?

#### 3.2.1 Which ML techniques were used?

To build a perspective of the role of ML in multi-omics integration, we investigated how it differed from ML’s role in data integration in general. In this view, we gathered 100 more papers, this time searching for ‘ML AND Integration’, omitting the multi-omics term. With these and our initial set, we recorded which ML techniques were used in each paper.

**Fig. 4.**
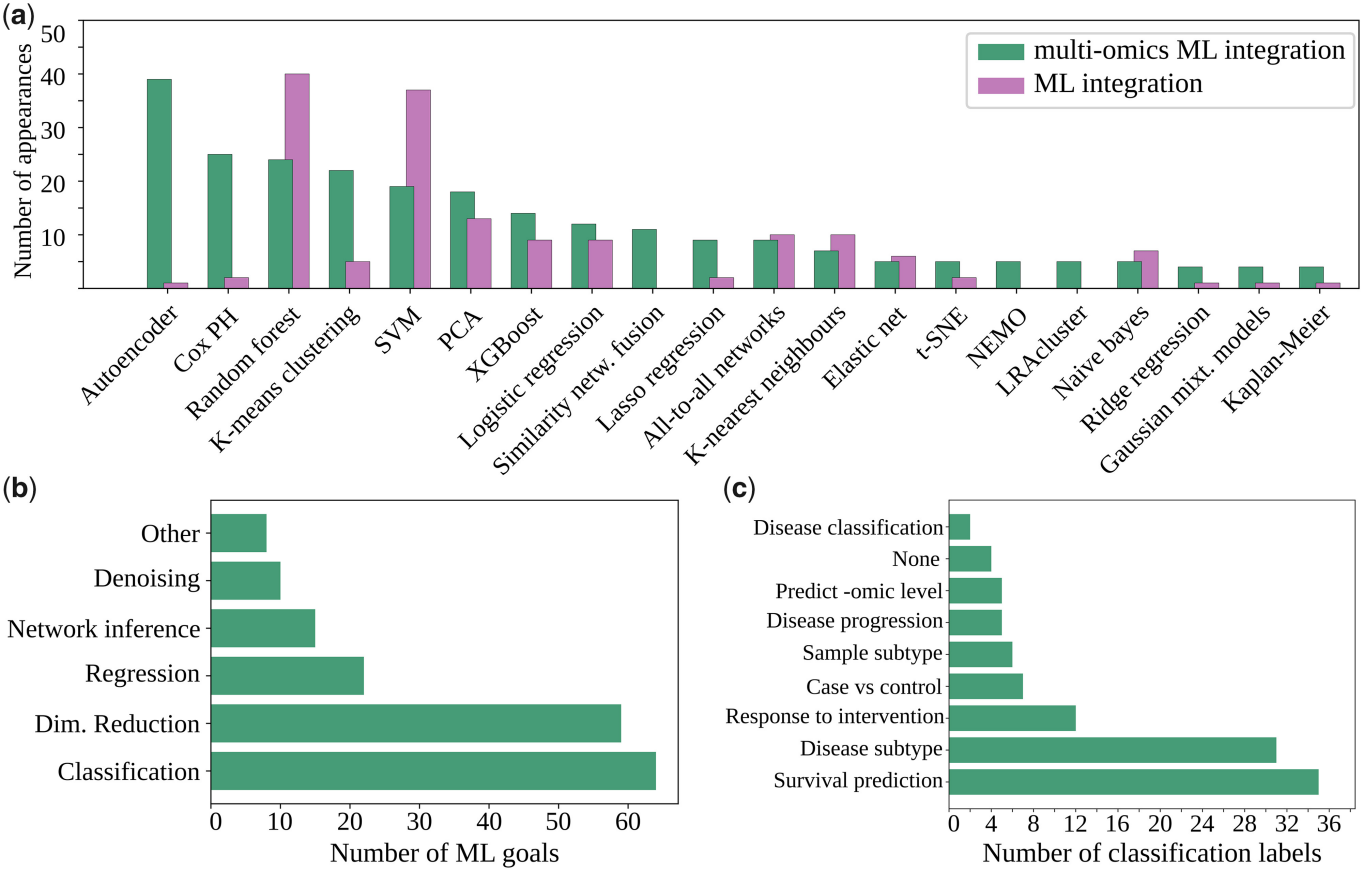
(**a**) Number of ML techniques being used more than once in the reviewed papers. The publications on ‘machine learning AND multi-omics AND integration’ are plotted in green, while the publications on ‘machine learning AND integration’ are plotted in purple. Significant differences were observed for autoencoders, and Cox proportional hazards (Cox PH) (Cox, 1972). Fisher’s exact test *P*-values of <0.0001 in both cases, satisfying the Bonferroni correction for this number of tests. Number in the reviewed multi-omics ML papers of ML goals (**b**) and labels used for classification (**c**)

In Figure 4a, we show the number of appearances of different ML techniques in the surveyed literature, omitting those that only appeared once. For the full results, see Supplementary Figure S2. Using Fisher’s exact test, we tested for a statistically significant difference in the number of appearances of each technique in the multi-omics group versus the general ML group. We found such differences in autoencoders and Cox PH where these were more common in the multi-omics group (see Supplementary Fig. S3).

#### 3.2.2 What were the goals of the ML application?

Classification (e.g. separating diseases into subtypes) was the most common goal in the reviewed papers (Fig. 4b). In some instances, this was used to discretize a regression problem. For example, several papers focused on survival prediction. Instead of predicting the number of years that a patient would survive (i.e. a regression task), the labels were ‘survival time > 5 years’ versus ‘survival time < 5 years’ (i.e. a classification task). Dimensionality reduction was the second most common goal, often applied before classification. Regression, network inference and denoising followed.

#### 3.2.3 What were the targets/labels of classification tasks?

The dominant category of labels was survival prediction (Fig. 4c), followed by disease/patient/organism subtyping and response to intervention. Other labels included disease progression and classification.

### 3.3 Can we explain trends using an analysis of the citation of papers since 2015?

**Fig. 5.**
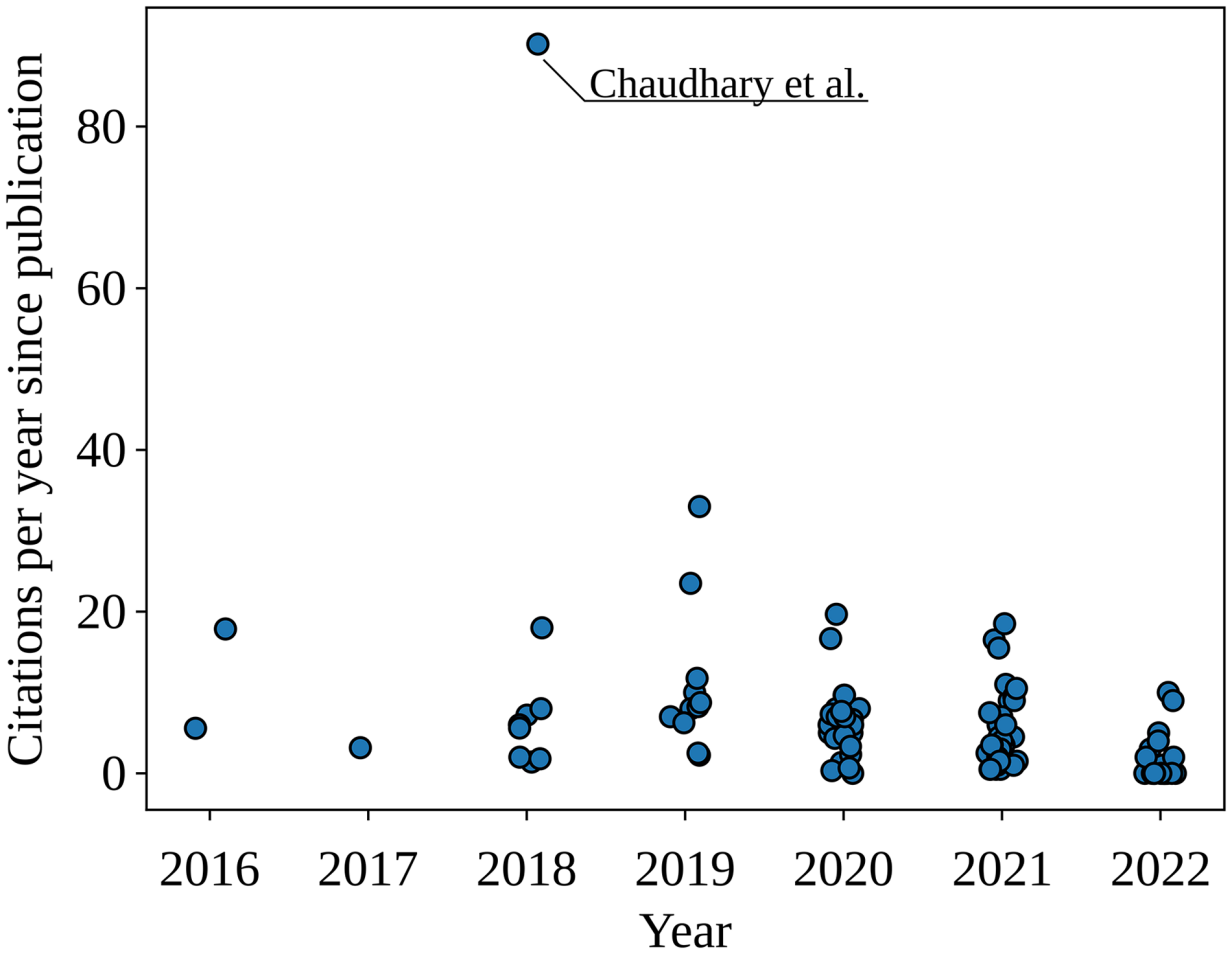
Number of citations per year, of papers published in different years

Figure 5 suggests that 2018 sparked an interest in this field, especially for autoencoders. In that year Chaudhary *et al.* (2018) was published and went on to become the most cited paper in this field. More details on this paper are included in the discussion.

## 4 Discussion

**Fig. 6.**
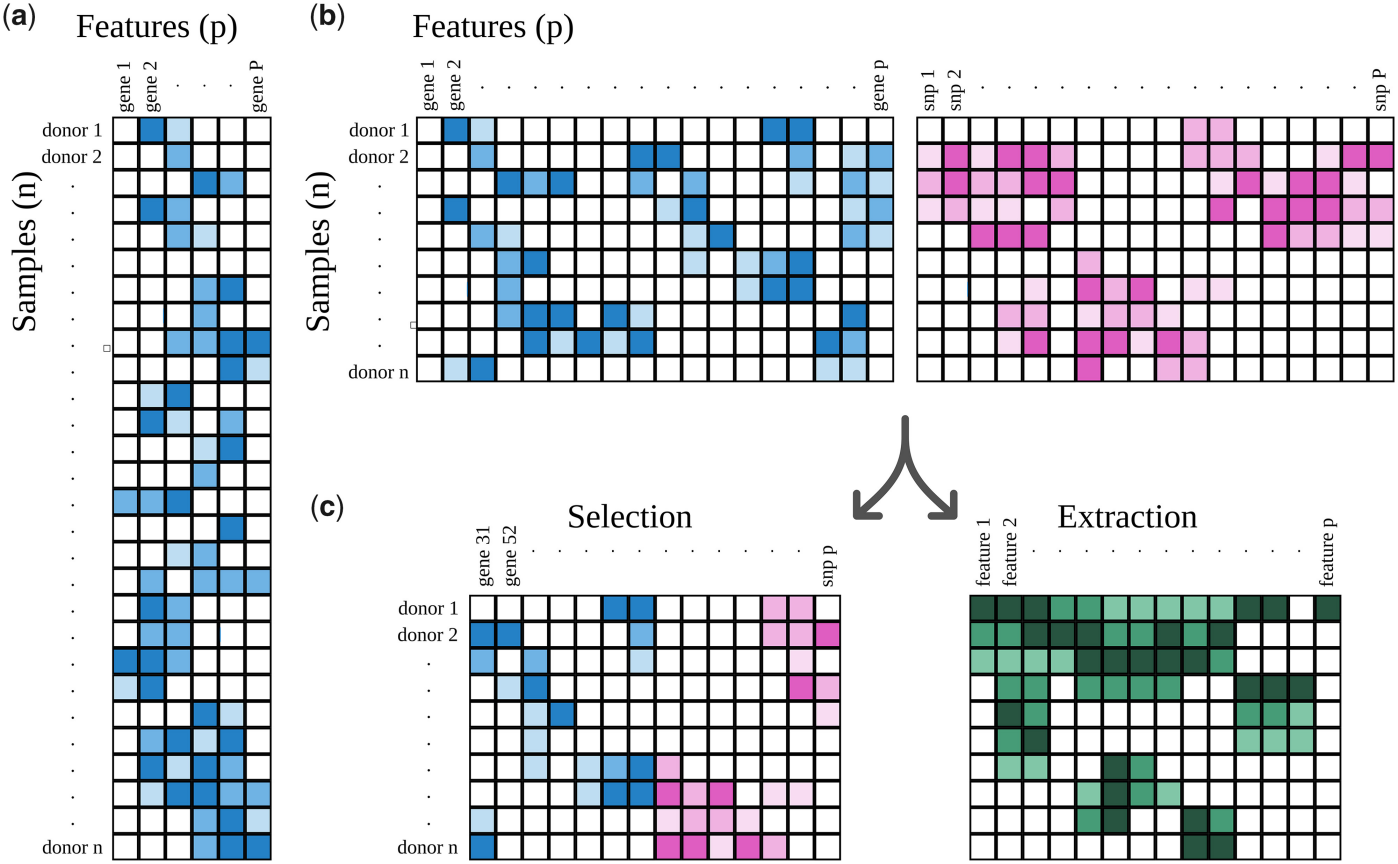
Shapes in datasets. (**a**) A dataset where *n* ≫ *P*, the ideal ‘shape’ for many ML techniques. (**b**) In multi-omics analyses, researchers face very wide datasets, where *n* ≪ *P*. (**c**) Feature selection and extraction are often used to reduce the number of features. In feature selection, a subset of the original features is kept. In feature extraction, features are merged and transformed into a smaller number of new ones

Multi-omics datasets often contain large numbers of features (*P*) for a relatively small number of samples (*n*). This has been described as the *n* ≪ *P* problem (see Fig. 6b). This is opposite of the ideal situation for many ML applications, where a dataset with *n* ≫* P* is considered ideal (Fig. 6a).

Mathematical analysis becomes more challenging in high-dimensional spaces. A typical problem with high dimensionality (large number of features) is data ‘sparsity’. Considering each datapoint as a coordinate, as the number of features increases (and therefore the number of data dimensions increases), the volume of the dimensional space spanned by the data points increases rapidly, such that the available data becomes sparse. This, in turn, makes inference and prediction particularly difficult unless large amounts of data points are available for analysis. This phenomenon is known as the ‘curse of dimensionality’, and the high heterogeneity of biological data amplifies this challenge for researchers.

We found that a vast share of ML multi-omic data integration approaches revolve around overcoming this ‘curse’. Researchers have to take steps to maximize the number of available samples and minimize the number of features. After that, they still tend to rely on models that are not too sensitive to having relatively few samples.

### 4.1 Reducing the number of features (*P*)

To minimize *P*, or the number of features, one can select a subset of more relevant features. Alternatively, one can use an algorithm that merges and transforms features in a smaller number of new ones, or one can apply a combination of the previous two approaches.

### 4.2 Feature selection

In computational modelling, any ability to leverage prior knowledge of the system into the model can be beneficial since appropriate prior assumptions can help to find an optimal model. This principle is often applied implicitly. For example, raw sequencing reads of RNAseq are not analysed as-is, but rather mapped onto gene transcripts as those have been defined in the latest genome assembly of a model organism. Similarly, the genome-wide signal of DNA methylation assays is typically discretized into a number of active/inactive methylation sites (Yuan *et al.*, 2019), since such sites can be considered functional units of gene regulation.

Beyond this discretization of the raw data, biological expertise can offer critical advantages through feature selection (see Fig. 6c) by discarding features with low probability to be relevant. For instance, Athreya *et al.* (2018) reduced over 7 million features consisting of SNPs and metabolites, to 65 predictor variables. They used a variety of reduction criteria including keeping SNPs that had a strong association with metabolite concentrations. Generally, some researchers will keep the most variable genes and discard those with consistently low activity levels. Epigenomic data can similarly be reduced by only considering the loci that are found near relevant genes or by looking at regions encompassing multiple methylation sites rather than looking at individual sites separately.

Feature selection can also be done using computational algorithms rather than biological expertise. One notably common application in multi-omics studies is to combine feature selection with survival analysis with the Cox PH model. The standard Cox PH model utilizes linear regression techniques for selecting informative features. This was the second most popular ML technique for multi-omics, but not so in the broader non-multi-omic field (Fig. 4a). The dominance of TCGA data, where survival analysis can often be applied, and the need of feature selection in the multi-omic field likely contributed to this disparity in popularity. Favourable characteristics of feature selection with Cox PH model applications include that the outcomes provide interpretable values representing the magnitude and direction of the effect of multiple features on survival. Furthermore, the Cox PH model is applicable also when the outcomes in survival data of the samples are incomplete, or *censored*, for example if a portion of patients are still alive and their survival time is thus unknown (Cox, 1972). This can help to extract information from the limited data, thus alleviating the low sample size issue.

### 4.3 Feature extraction

Even after feature selection, most multi-omic datasets will have a high ratio of features/samples and will necessitate further reduction of the count of features, for example, by feature extraction. Principal component analysis (PCA), a popular technique in biological research, is an example of such a feature-extracting technique. Feature extraction (see Fig. 6c) is the process of condensing features (e.g. gene activity levels) into a user-defined number of new features. In PCA, these new features are the principal components, the top two of which are typically plotted on the classic PCA plot. A downside of PCA is that it only enables linear transformation of the data, whereas relationships in biology are rarely linear in real life (Zuin, 2022). Furthermore, a degree of explainability is lost from the features as it is not always easy to explain what a principal component exactly is.

Another example of feature extraction are autoencoders. These neural networks run input data through a series of layers of a neural network, with one of these being a smaller ‘bottleneck’ layer. As an example, Chaudhary *et al.* (2018) used a network with three layers of 500, 100 and 500 units, respectively. This middle bottleneck layer produces the extracted features which can then be used for further analysis. Aspects of this paper have been echoed in many subsequent papers, such as using autoencoders, the TCGA database and K-means clustering.

An advantage of autoencoders is that by adding several layers with non-linear activation functions, the technique can model complex nonlinear functions. Autoencoders were more popular for multi-omic ML data integration than for general ML data integration.

Autoencoders take in the original inputs, compress them into a lower dimensional representation and then reconstruct the inputs minimizing the difference between original and reconstructed inputs (i.e. ‘reconstruction loss’). Denoising is particularly pertinent to multi-omics. Here ‘noise’ can be thought of as something that randomly changes pixel values of an image, so that the resulting image is corrupted. Denoising autoencoders (Vincent *et al.*, 2008) add to the autoencoder framework by corrupting (adding noise to) the original inputs. An example is Seal *et al.* (2020), which added a randomly drawn number to each input. The aim of this is that the model learns to create a lower- dimensional representation that retains the most important aspects of the original information.

The success of autoencoders may largely be thanks to the application of the backpropagation (Rumelhart *et al.*, 1986) algorithm. This is an optimization algorithm that has proven widely effective in adjusting ML model parameters towards specific goals (e.g. minimizing reconstruction loss). In addition, autoencoders are relatively easy to use.

### 4.4 Increasing the number of samples (*n*)

An alternative to producing a problem-specific dataset and maximizing n by deeper sampling, is to turn to publicly available data. Motivations for using public data could be either lack of resources or because of different research interests. Researchers in the papers we surveyed overwhelmingly relied on public databases of multi-omic data, mainly, TCGA.

TCGA contains approximately 10 000 samples across 33 human cancer types (Tomczak, 2015) in a well maintained, sophisticated portal with an application programming interface (API). This high number of datasets combined with its ease of use have made it a favourite source for researchers. A possible limitation of the field's reliance on this dataset is its focus on cancer. Cancer is characterized by highly dysregulated metabolism and aberrant signalling pathways (Cairns, 2011), which may lead to relatively easily identifiable -omics signals. This may not be congruent with other fields, such as agriculture or nutrition where small interventions are made (e.g. changing feed), and which may generate more subtle -omics signals (Edmunds *et al.*, 2012). Another disadvantage of the widespread use of this database is that it does not contain certain types of omics, such as ATACseq. If the multi-omics community relied too heavily on TCGA, it may bias future research towards the -omics data types contained in the TCGA dataset. On the other hand, Figure 3 shows that TCGA and other datasets are fairly comparable (although statistically different in number of features), TCGA could be generally representative of other datasets.

Multi-omic datasets often include non-overlapping data, i.e. where not all -omics data are obtained for each sample. This can lead to a significant decrease in viable samples, reducing statistical power for the analysis. For example, in Zhang (2018), data from 407 neuroblastoma patients were used. Of those, 380 had copy number alteration data, 217 had gene expression data, but only 190 had both and these 190 were subsequently used in the integration analysis.

### 4.5 After reshaping the data

Feature extraction techniques comprised the first, second and fifth most popular techniques. The rest were techniques that perform ML tasks such as classification, clustering, regression and inferring networks among other goals.

Several papers used ML to perform classification, primarily for survival time prediction. There were varied reasons for using survival time as a label, ranging from elucidating mechanisms of tumour progression (Poirion, 2021) to improving treatment decisions (Mitchel, 2020).

### 4.6 New ML developments of potential use in multi-omics

Looking forward, there have been several developments in ML in the last five years and their implications for multi-omics research are yet to be fully realized. These include a rekindled interest in understanding causality in systems, graph-based models and transformers.

Multi-omics research should, in theory, lend itself to causal interpretation, according to the central dogma of biology (Crick, 1958, 1970) where DNA → RNA → Protein. Inferring causality in systems is important, particularly when planning to intervene in the system (e.g. manipulating DNA, RNA or protein to change a phenotype) (Barsi, 2021). A recent multi-omics paper (Zenere, 2022) applied graphical models (Pearl, 1988) to construct ‘protein coding units’ consisting of epigenomics, transcriptomics and proteomics data. The general pattern of causal inference in research is to formulate potential causal relationships from the literature or another source of prior knowledge and then test these relationships against a given dataset (Baker, 2022). Dugourd *et al.* (2021) took this approach by constructing prior knowledge networks from public datasets to identify potential causal relationships and then tested which of these were reflected in the data. With their findings they were able to make inferences about potential cancer treatment targets. Causal discovery, i.e. learning the causal relations that define the graph structure, without imposing prior knowledge is more difficult. One way of approaching this, however, is to look for relations that are stable across multiple experimental conditions. An example of this Invariant Causal Prediction principle is (Meinshausen, 2016) who looked at gene perturbation experiments. See also (Peters, 2016) for further mathematical details. The structural assumption on the causal relations can also be replaced by distributional assumptions, e.g. as in LiNGAM (linear non-gaussian acyclic model) (Shohei, 2006) or CAM (causal additive model) (Bühlmann, 2014). However, since reliable causal discovery requires more samples than classical association and prediction models, these methods are still uncommon in the multi-omics literature.

Graph neural networks are a class of deep learning approaches used for processing large amounts of data represented by graphs, in order to learn tasks from such graphs (Muzio *et al.*, 2021). Among these, graph convolutional networks are particularly promising: they use the convolutional layers architecture (LeCun, 1995) to learn important features from input graphs, which can be used for various tasks, including prediction (Singha, 2020) or classification (Zitnik, 2018). In multi-omics, graph convolutional neural networks have had various applications. For example, (Peng *et al.*, 2022) used them for predicting cancer drug response, and (Wang, 2021; Zhang, 2021) used them to do biomedical classifications (e.g. Alzheimer’s disease diagnosis and pan-cancer classification, respectively). As much as convolutional architectures have excelled in fields such as vision, a newer technique, the attention mechanism (Bahdanau, 2014), has shown to be superior in various settings (Vaswani, 2017). And so, it is not surprising that graph-attention-based models have also been used in multi-omics research, for example (Xing, 2022), which performed disease classification and survival prediction.

The attention mechanism is also at the heart of what is today probably the most powerful deep learning technique—transformers. Transformers are deep learning models that employ the attention mechanism instead of convolutional or fully connected layers. They gained traction in the field of natural language processing (NLP) where they have outperformed recurrent neural networks (RNN) and long short-term memory (LSTM) models and are quickly becoming the state-of-the-art solution in many fields, for example object detection (Zhang, 2022).

Transformers are used to process sequential data, which is often well suited for biological applications. An example is AlphaFold (Jumper, 2021) which has revolutionized the field of protein folding modelling, and which relies, in part, on transformers. Another example is the ‘Big Bird’ model (Zaheer, 2020), an iteration on the BERT model (Devlin, 2018). BigBird was shown to work both on NLP but also on genomic tasks where it achieved improved performance on downstream tasks such as promoter-region and chromatin profile prediction. Furthermore, transformers can be pre-trained in an unsupervised way, an approach that could help ameliorate the ‘few samples’ problem by incorporating prior biological knowledge into the model.

Although computationally expensive, their accuracy has already propelled transformers to become state of the art in a number of biological applications (Avsec, 2021; Jumper, 2021).

## 5 Conclusion

Multi-omics datasets tend to contain orders of magnitude more features than samples, making dimensionality reduction a key issue in their analysis. Typical steps included manual feature selection, followed by algorithmic selection and/or extraction. Autoencoders and Cox PH were commonly used. Causal models, graph neural networks and transformers are some promising approaches for the field.

ML was most often used in classification problems, where tools like random forests and SVM were most commonly used. Their low barrier to entry, adaptability to many kinds of data types, and ability to work with relatively few samples are possible reasons for their popularity. These are both not new techniques, which may suggest that there is a gap for classification techniques that cope well with highly dimensional data and a low number of samples.

The dominance of TCGA as a source of data in the multi-omics ML integration field, highlights the impact that such a database can have. Datasets that are easy to discover and use propel innovation by allowing fast iterations over different techniques. Curating datasets and goals for ML competitions could accelerate this even further. Perhaps a good direction for the field would be to produce more datasets like TCGA, but for different model organisms and contexts (e.g. agricultural crops under different interventions). Major projects like Holofood (2019) show that researchers and funders are maybe already aware of the importance of such datasets.

## Supplementary Material

btad021_Supplementary_Data
