# Supplementary material for: Dealing with dimensionality: the application of machine learning to multi-omics data
Source: Bioinformatics. 2023 Jan 13;39(2):btad021. doi: 10.1093/bioinformatics/btad021 (PMC9907220; doi:10.1093/bioinformatics/btad021)
Supplement: btad021_Supplementary_Data [file btad021_supplementary_data.pdf]

Supplementary Material

Eligibility criteria

We considered research papers in English that were published in 2015 and afterwards, including pre-prints. We excluded reviews, opinion pieces, conference proceedings, non-indexed publications, and publications that did not use ML to integrate multi-omics data.

Information sources

We used the Google Scholar database, covering the years 2015 to present. The date we last accessed it was 4 July 2022. The year 2015 marked a new era in ML research, since the release of software libraries like TensorFlow (Abadi 2016) and Keras (Chollet et al., 2015 available at https://github.com/fchollet/keras) in 2015 and Pytorch (Paszke 2019) in 2016 allowed for easier application of ML models on GPUs. Following this, the application of ML methods has rapidly gained popularity.

Search

Our full search strategy for the Google Scholar database was as follows:

1) Set a custom time range to 2015-present.
2) Search for "machine learning AND multi-omics AND integration".
3) Order results by the Google Scholar "relevance" algorithm, which seems to be heavily influenced by citation count (Beel 2009)[1] . In our experience, it seems to also prioritise more recent papers.

[1] While this may exclude some newer papers which have not had as much time to gather citations, we argue that it is a worthy trade-off for finding the most influential papers in the field.

4) Open each result, check if it fits our eligibility criteria.
5) Obtain answers to our review questions.
6) Stop when we reach 100 eligible publications.

Synthesis of results

Publications were individually assessed by the researchers and data was collected per research question. These were then manually collated. Following this, we went through the collated results to extract patterns/ trends/ clusters of paper types, using plots when applicable. All processed data and plotting scripts are available in the project's Gitlab repository. gitlab.com/polavieja_lab/ml_multi-omics_review , or in Zenodo doi.org/10.5281/zenodo.7361807

Data charting process

For each research question we created a spreadsheet. In the leftmost column we entered the names of our 100 papers. In the next column we entered the answer we had found for the research question after reading the paper. We categorised each of the papers sequentially by entering a '1' in the relevant column to the right. As we went through the papers, we added further category columns as necessary. After completing this process, we used python libraries, primarily pandas and seaborn (Pandas dev. team 2020, Waskom 2021), to structure the data and create the plots for this report.

Data items/ characteristics of sources of evidence

For each paper, we gathered answers to our research questions, namely:

**Research question**	**Data gathered**
What was the goal of the ML application?	Which broad ML goal did the study fit under, out of:<br>● Classification<br>● Regression<br>● Dimensionality reduction<br>● Network inference<br>● Other
Which ML techniques were used?	Which type of technique was used, without using categories we simply noted each machine learning technique used in each paper.
Was a specific dataset used?	We noted which dataset was used and whether this was:<br>● The Cancer Genome Atlas (TCGA)<br>● Other

Which omics types were used?	We categorised the omics types into:<br>● Transcriptomics, including:<br>● RNA-Seq<br>● MiRNA-Seq<br>● Long non-coding RNA<br>● Whole transcriptome<br>● Epigenomics, including:<br>● Hi-C<br>● Chip-Seq<br>● DNA methylation<br>● Gene methylation<br>● CpG<br>● Chromatin accessibility<br>● Open chromatin regions<br>● Genomics, including:<br>● Copy number variation<br>● Copy burden<br>● Copy aberration<br>● Copy alteration<br>● Whole genome, SNPs<br>● Mutation<br>● Transcription factor binding sites<br>● Metabolomics

	- Proteomics - Protein expression - Reverse phase protein array - Metagenomics, e.g. 16s - Other, including: - Fluxome - QTX - Histopathological features - Microarray - Clinical - Flow cytometry - Imaging
What was the shape of the data?	Where multiple studies were performed within a paper, we took the maximum.

What labels were used?	We categorised the type of label used into:<br><br>• Survival prediction<br>• Disease subtype<br>• Patient/ organism subtype<br>• Predict response to interventions<br>• Individual omic level<br>• None<br>• Disease progression<br>• Clustering to find survival subtypes<br>• Known association of omic to disease<br>• Case vs control<br>• Disease A vs disease B

Selection and results of sources of evidence

The full list of the publications included in this review can be found in the references. All tables with specific responses to the research question for each are included in the GitLab repository: https://gitlab.com/polavieja_lab/ml_multi-omics_review/

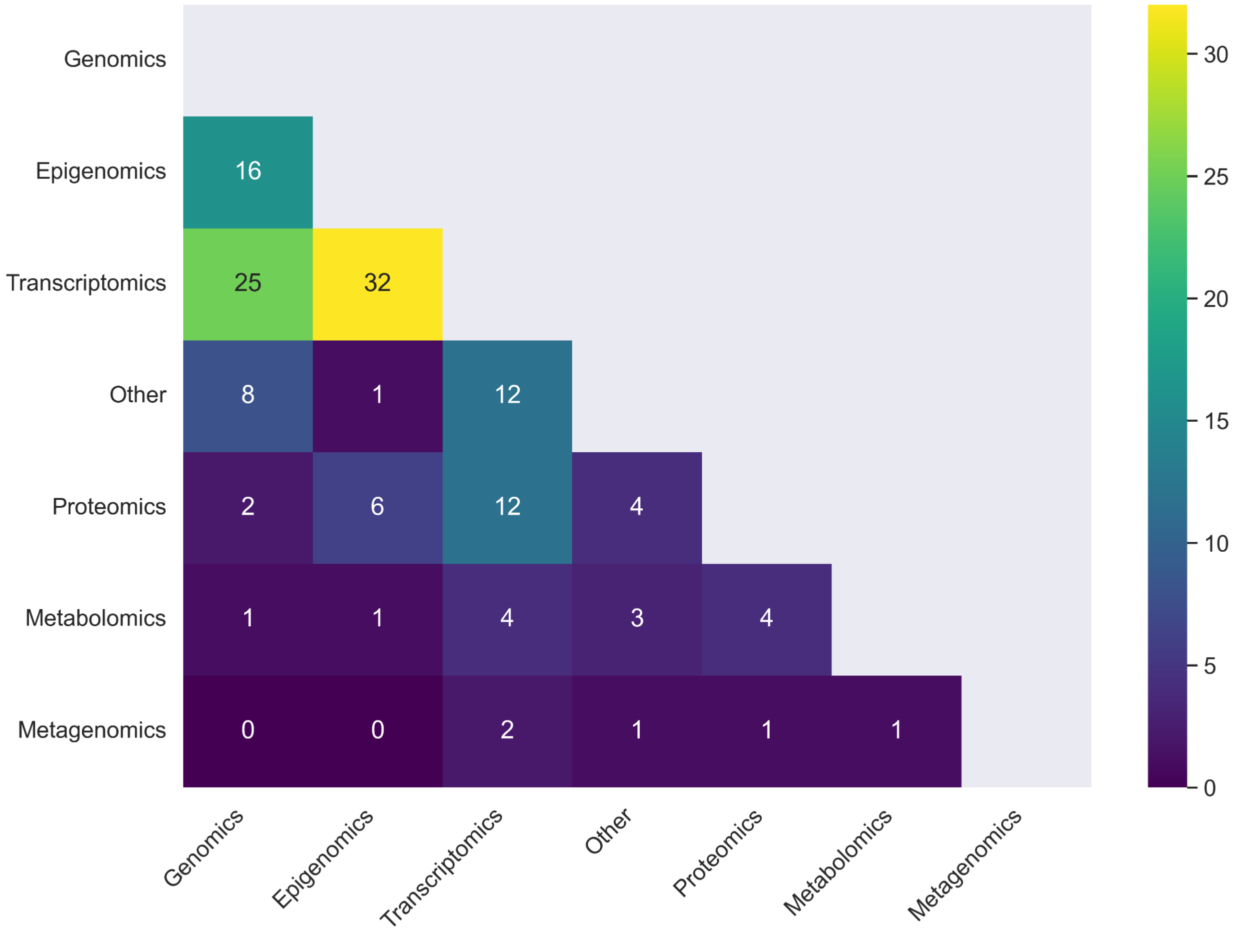

Supplementary Figure 1. Distribution of the number of -omics type combinations used across papers, excluding TCGA.

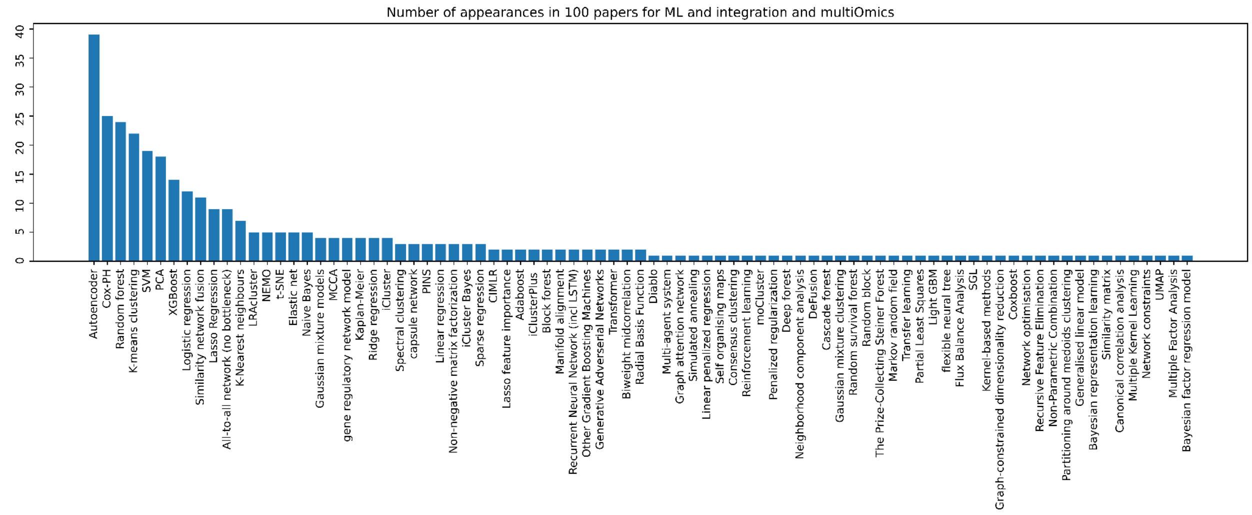

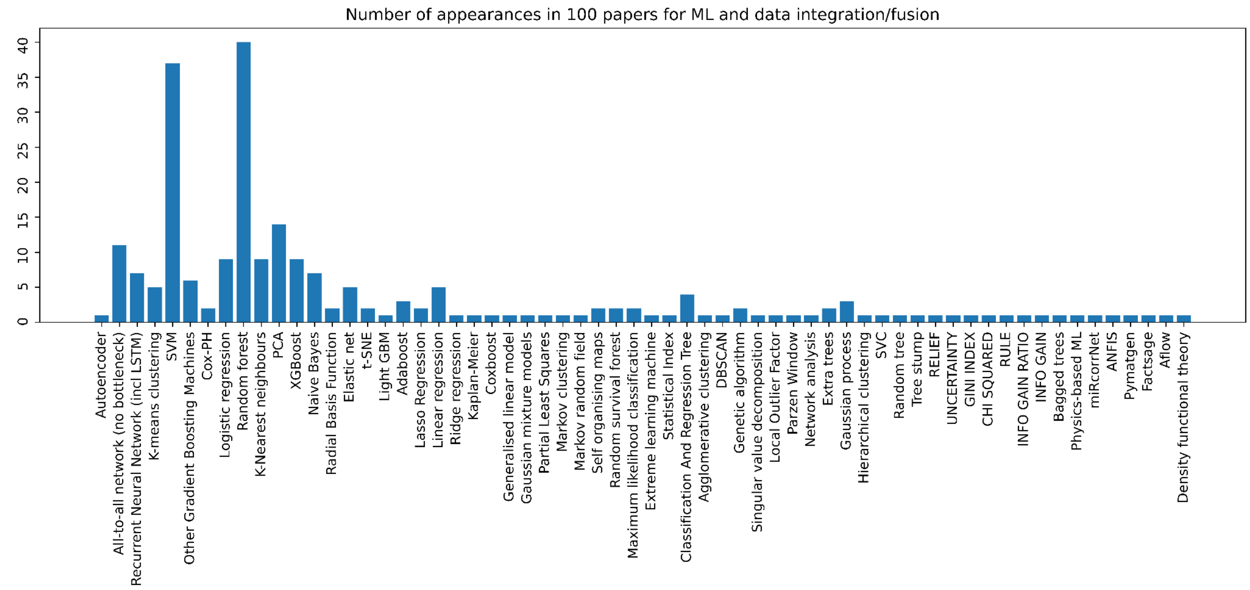

Supplementary figure 2. Top: all ML AND Integration AND multi-omics techniques, bottom: all ML AND Integration techniques.

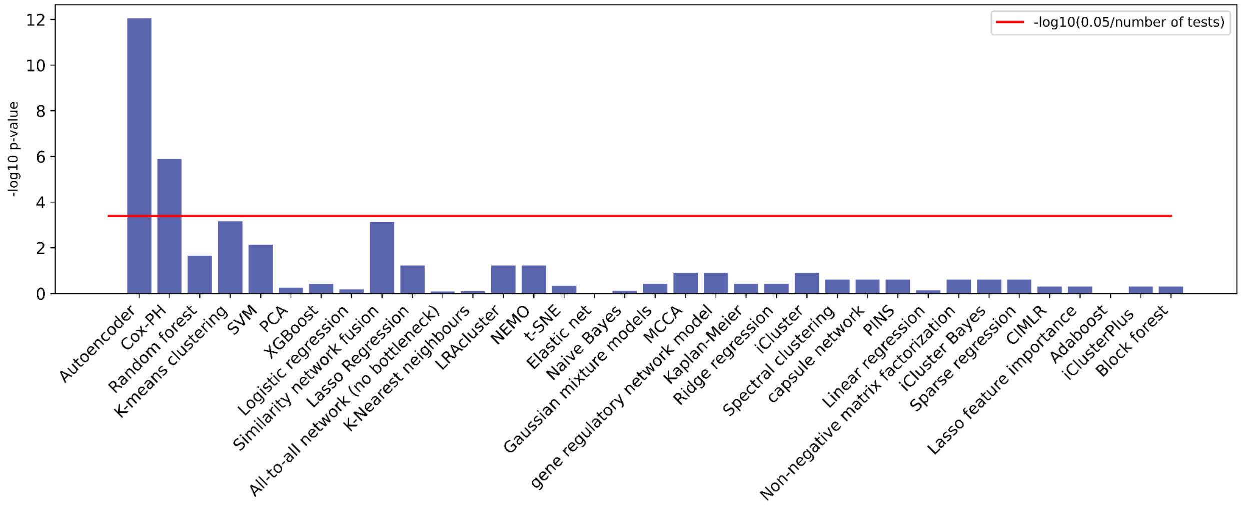

Supplementary figure 3. Fisher’s exact test showing which techniques are used significantly more often in “ML AND Integration AND multi-omics” versus in “ML AND Integration techniques”

References

Abadi M et al. (2016) Tensorflow: Large-scale machine learning on heterogeneous distributed systems. arXiv preprint arXiv:1603.04467.

Beel J, Gipp B (2009) Google Scholar's ranking algorithm: an introductory overview. Proceedings of the 12th international conference on scientometrics and informetrics (ISSI'09). Vol. 1.

Chollet F et al. (2015) Keras. https://github.com/fchollet/keras.

Pandas dev. team (2020) "pandas-dev/pandas: Pandas", Zenodo, 10.5281/zenodo.3509134

Paszke A et al. (2019) PyTorch: An Imperative Style, High-Performance Deep Learning Library. Advances in Neural Information Processing Systems, 32, 8024–8035

Waskom ML (2021) Seaborn: statistical data visualization. Journal of Open Source Software, 6.60: 3021.

Lists of the reviewed papers

The 100 papers that were selected from the "Machine Learning AND multi-omics AND integration" search.

Albaradei S et al. (2021) MetaCancer: A deep learning-based pan-cancer metastasis prediction model developed using multi-omics data. Computational and Structural Biotechnology Journal 19, 4404-4411.

Althubaiti S et al. (2021) DeepMOCCA: A pan-cancer prognostic model identifies personalized prognostic markers through graph attention and multi-omics data integration. bioRxiv.

Baek B et al. (2020) Prediction of survival and recurrence in patients with pancreatic cancer by integrating multi-omics data. Sci Rep 10(1).

Bhak Y et al. (2019) Depression and suicide risk prediction models using blood-derived multi-omics data. Transl Psychiatry 9(1).

Bica I et al. (2018) Multi-omics data integration using cross-modal neural networks. In ESANN.

Cao K et al. (2020) Unsupervised topological alignment for single-cell multi-omics integration. Bioinformatics 36(Supplement_1), i48-i56.

Chai H et al. (2019) Integrating multi-omics data through deep learning for accurate cancer prognosis prediction. bioRxiv 807214

Chai H et al. (2021) Integrating multi-omics data through deep learning for accurate cancer prognosis prediction. Computers in Biology and Medicine 134, 104481.

Chaudhary K et al. (2018) Deep Learning–Based Multi-Omics Integration Robustly Predicts Survival in Liver Cancer. Clinical Cancer Research 24(6), 1248-1259.

Chierici M et al. (2020) Integrative Network Fusion: A Multi-Omics Approach in Molecular Profiling. Front. Oncol. 10.

Chung N et al. (2019) Unsupervised classification of multi-omics data during cardiac remodeling using deep learning. Methods 166, 66-73.

Du L et al. (2022) Uncertainty-aware dynamic integration for multi-omics classification of tumors. J Cancer Res Clin Oncol.

Duan R et al. (2021) Evaluation and comparison of multi-omics data integration methods for cancer subtyping. PLoS Comput Biol 17(8), e1009224.

El-Manzalawy Y (2018) CCA based multi-view feature selection for multi-omics data integration. 2018 IEEE Conference on Computational Intelligence in Bioinformatics and Computational Biology (CIBCB).

EL-Manzalawy Y et al. (2018) Min-redundancy and max-relevance multi-view feature selection for predicting ovarian cancer survival using multi-omics data. BMC Med Genomics 11(S3).

El-Nabawy A et al. (2021) A Cascade Deep Forest Model for Breast Cancer Subtype Classification Using Multi-Omics Data. Mathematics 9(13), 1574.

Francescatto M et al. (2018) Multi-omics integration for neuroblastoma clinical endpoint prediction. Biol Direct 13(1).

Franco E et al. (2021) Performance Comparison of Deep Learning Autoencoders for Cancer Subtype Detection Using Multi-Omics Data. Cancers 13(9), 2013.

Fu Y et al. (2020) A gene prioritization method based on a swine multi-omics knowledgebase and a deep learning model. Commun Biol 3(1).

Gillenwater L et al. (2021) Multi-omics subtyping pipeline for chronic obstructive pulmonary disease. PLoS ONE 16(8), e0255337.

Guo L et al. (2020) Deep learning-based ovarian cancer subtypes identification using multi-omics data. BioData Mining 13(1).

Hajiramezanali E et al. (2020). BayReL: Bayesian relational learning for multi-omics data integration. Advances in Neural Information Processing Systems, 33, 19251-19263.

Herrmann M et al. (2020) Large-scale benchmark study of survival prediction methods using multi-omics data. Briefings in Bioinformatics 22(3).

Hira M et al. (2021) Author Correction: Integrated multi-omics analysis of ovarian cancer using variational autoencoders. Sci Rep 11(1).

Holzscheck N et al. (2020) Multi-omics network analysis reveals distinct stages in the human aging progression in epidermal tissue. Aging 12(12), 12393-12409.
Hu Y et al. (2022) Classifying the multi-omics data of gastric cancer using a deep feature selection method. Expert Systems with Applications 200, 116813.
Huang E et al. (2021) Deep Learning for Integrated Analysis of Insulin Resistance with Multi-Omics Data. JPM 11(2), 128.
Huang L et al. (2019) Driver network as a biomarker: systematic integration and network modeling of multi-omics data to derive driver signaling pathways for drug combination prediction. Bioinformatics 35(19), 3709-3717.
Huang Z et al. (2019) SALMON: Survival Analysis Learning With Multi-Omics Neural Networks on Breast Cancer. Front. Genet. 10.
Jin T et al. (2021) scGRNom: a computational pipeline of integrative multi-omics analyses for predicting cell-type disease genes and regulatory networks. Genome Med 13(1).
Joyce J et al. (2021) Multi-omics driven predictions of response to acute phase combination antidepressant therapy: a machine learning approach with cross-trial replication. Transl Psychiatry 11(1).
Kanapeckaitė A et al. (2021) Insights into therapeutic targets and biomarkers using integrated multi-'omics' approaches for dilated and ischemic cardiomyopathies. Integrative Biology 13(5), 121-137.
Kaur H et al. (2021) Gut microbiome-mediated epigenetic regulation of brain disorder and application of machine learning for multi-omics data analysis. Genome 64(4), 355-371.
Kim M et al. (2016) Multi-omics integration accurately predicts cellular state in unexplored conditions for Escherichia coli. Nat Commun 7(1).
Lee C et al. (2021) A variational information bottleneck approach to multi-omics data integration. In International Conference on Artificial Intelligence and Statistics (pp. 1513-1521). PMLR.
Lee T et al. (2020) Incorporating deep learning and multi-omics autoencoding for analysis of lung adenocarcinoma prognostication. Computational Biology and Chemistry 87, 107277.
Lemsara A et al. (2020) PathME: pathway based multi-modal sparse autoencoders for clustering of patient-level multi-omics data. BMC Bioinformatics 21(1).
Leng D et al. (2022) A benchmark study of deep learning-based multi-omics data fusion methods for cancer. Genome Biol 23(1).
Lewis J et al. (2021) Integration of machine learning and genome-scale metabolic modeling identifies multi-omics biomarkers for radiation resistance. Nat Commun 12(1).
Li F et al. (2022) Effects of Multi-Omics Characteristics on Identification of Driver Genes Using Machine Learning Algorithms. Genes 13(5), 716.
Lin Y et al. (2020) Classifying Breast Cancer Subtypes Using Deep Neural Networks Based on Multi-Omics Data. Genes 11(8), 888.
Ma B et al. (2020) Diagnostic classification of cancers using extreme gradient boosting algorithm and multi-omics data. Computers in Biology and Medicine 121, 103761.
Ma T et al. (2019) Integrate multi-omics data with biological interaction networks using Multi-view Factorization AutoEncoder (MAE). BMC Genomics 20(S11).
Malik V et al. (2021) Deep learning assisted multi-omics integration for survival and drug-response prediction in breast cancer. BMC Genomics 22(1).

Mitchel J et al. (2019) A Translational Pipeline for Overall Survival Prediction of Breast Cancer Patients by Decision-Level Integration of Multi-Omics Data. 2019 IEEE International Conference on Bioinformatics and Biomedicine (BIBM).

Oh M et al. (2020) DRIM: A Web-Based System for Investigating Drug Response at the Molecular Level by Condition-Specific Multi-Omics Data Integration. Front. Genet. 11.

Pan X et al. (2022) Deep learning based on multi-omics integration identifies potential therapeutic targets in breast cancer. bioRxiv

Pan X et al. (2022) i-Modern: Integrated multi-omics network model identifies potential therapeutic targets in glioma by deep learning with interpretability. Computational and Structural Biotechnology Journal 20, 3511-3521.

Patel-Murray N et al. (2020) A Multi-Omics Interpretable Machine Learning Model Reveals Modes of Action of Small Molecules. Sci Rep 10(1).

Peng C et al. (2020) Capsule Network Based Modeling of Multi-omics Data for Discovery of Breast Cancer-Related Genes. IEEE/ACM Trans. Comput. Biol. and Bioinf. 17(5), 1605-1612.

Planell N et al. (2021) STATegra: Multi-Omics Data Integration – A Conceptual Scheme With a Bioinformatics Pipeline. Front. Genet. 12.

Poirion O (2018) Deep learning data integration for better risk stratification models of bladder cancer. AMIA Summits on Translational Science Proceedings, 2018, 197.

Poirion O et al. (2019) DeepProg: an ensemble of deep-learning and machine-learning models for prognosis prediction using multi-omics data. Genome medicine, 13(1), 1-15.

Rabaglino M et al. (2021) Application of multi-omics data integration and machine learning approaches to identify epigenetic and transcriptomic differences between in vitro and in vivo produced bovine embryos. PLoS ONE 16(5), e0252096.

Rakshit S et al. (2018) Deep Learning for Integrated Analysis of Breast Cancer Subtype Specific Multi-omics Data. TENCON 2018 - 2018 IEEE Region 10 Conference.

Rodosthenous T et al. (2020) Integrating multi-OMICS data through sparse canonical correlation analysis for the prediction of complex traits: a comparison study. Bioinformatics 36(17), 4616-4625.

Saha S et al. (2022) Integrating Two Deep Learning Models to Identify Gene Signatures in Head and Neck Cancer from Multi-Omics Data. Applied Smart Health Care Informatics, 67-81.

Seal D et al. (2020) Estimating gene expression from DNA methylation and copy number variation: A deep learning regression model for multi-omics integration. Genomics 112(4), 2833-2841.

Sharifi-Noghabi H et al. (2019) MOLI: multi-omics late integration with deep neural networks for drug response prediction. Bioinformatics 35(14), i501-i509.

Sherill-Rofe D et al. (2022) Multi-omics data integration analysis identifies the spliceosome as a key regulator of DNA double-strand break repair. NAR Cancer 4(2).

Singh A et al. (2016) DIABLO-an integrative, multi-omics, multivariate method for multi-group classification. BioRxiv, 067611.

Singh R et al. (2020) Unsupervised manifold alignment for single-cell multi-omics data. Proceedings of the 11th ACM International Conference on Bioinformatics, Computational Biology and Health Informatics.

Tabakhi S et al. (2022) Multi-agent Feature Selection for Integrative Multi-omics Analysis. 2022 44th Annual International Conference of the IEEE Engineering in Medicine & Biology Society (EMBC).

Takahashi S et al. (2020) Predicting Deep Learning Based Multi-Omics Parallel Integration Survival Subtypes in Lung Cancer Using Reverse Phase Protein Array Data. Biomolecules 10(10), 1460.

Tong D et al. (2020) Improving prediction performance of colon cancer prognosis based on the integration of clinical and multi-omics data. BMC Med Inform Decis Mak 20(1).

Tong L et al. (2020) Deep learning based feature-level integration of multi-omics data for breast cancer patients survival analysis. BMC Med Inform Decis Mak 20(1).

Tong L et al. (2021) Integrating multi-omics data by learning modality invariant representations for improved prediction of overall survival of cancer. Methods 189, 74-85.

Uyar B et al. (2021) Multi-omics and deep learning provide a multifaceted view of cancer. bioRxiv

Viaud G et al. (2022) Representation Learning for the Clustering of Multi-Omics Data. IEEE/ACM Trans. Comput. Biol. and Bioinf. 19(1), 135-145.

Wang T et al. (2020) MORONET: Multi-omics Integration via Graph Convolutional Networks for Biomedical Data Classification. bioRxiv, 10(2020.07), 02-184705.

Wang T et al. (2021) MOGONET integrates multi-omics data using graph convolutional networks allowing patient classification and biomarker identification. Nat Commun 12(1).

Wang W et al. (2021) DeFusion: a denoised network regularization framework for multi-omics integration. Briefings in Bioinformatics 22(5).

Wang X et al. (2021) Contrastive cycle adversarial autoencoders for single-cell multi-omics alignment and integration. arXiv preprint arXiv:2112.03266.

Wang Y et al. (2021) DeepDRK: a deep learning framework for drug repurposing through kernel-based multi-omics integration. Briefings in Bioinformatics 22(5).

Wei Z et al. (2022) Deep Learning-Based Multi-Omics Integration Robustly Predicts Relapse in Prostate Cancer. Front. Oncol. 12.

Wissel D et al. (2021) Hierarchical autoencoder-based integration improves performance in multi-omics cancer survival models through soft modality selection. bioRxiv.

Xie G et al. (2019) Group Lasso Regularized Deep Learning for Cancer Prognosis from Multi-Omics and Clinical Features. Genes 10(3), 240.

Xu C et al. (2020) AutoGenome V2: New Multimodal Approach Developed for Multi-Omics Research. bioRxiv.

Xu H et al. (2021) A network embedding based method for partial multi-omics integration in cancer subtyping. Methods 192, 67-76.

Xu J et al. (2019) A hierarchical integration deep flexible neural forest framework for cancer subtype classification by integrating multi-omics data. BMC Bioinformatics 20(1).

Yang B et al. (2022) Deep structure integrative representation of multi-omics data for cancer subtyping. Bioinformatics 38(13), 3337-3342.

Yang H et al. (2021) Subtype-GAN: a deep learning approach for integrative cancer subtyping of multi-omics data. Bioinformatics 37(16), 2231-2237.

Yang H et al. (2022) From multi-omics data to the cancer druggable gene discovery: a novel machine learning-based approach. bioRxiv.

Yang H et al. (2022) Subtype-Former: a deep learning approach for cancer subtype discovery with multi-omics data. arXiv preprint arXiv:2207.14639.
Yang H et al. (2022) Trans-Driver: a deep learning approach for cancer driver gene discovery with multi-omics data. bioRxiv.
Yang Y et al. (2021) A multi-omics-based serial deep learning approach to predict clinical outcomes of single-agent anti-PD-1/PD-L1 immunotherapy in advanced stage non-small-cell lung cancer. American journal of translational research, 13(2), 743-756.
Yang Y et al. (2022) MDICC: novel method for multi-omics data integration and cancer subtype identification. Briefings in Bioinformatics 23(3).
Yoosuf N et al. (2021) Early prediction of clinical response to anti-TNF treatment using multi-omics and machine learning in rheumatoid arthritis. Rheumatology 61(4), 1680-1689.
Yuan L et al. (2019) Integration of Multi-Omics Data for Gene Regulatory Network Inference and Application to Breast Cancer. IEEE/ACM Trans. Comput. Biol. and Bioinf. 16(3), 782-791.
Yuan L et al. (2021) A machine learning framework that integrates multi-omics data predicts cancer-related LncRNAs. BMC Bioinformatics 22(1).
Zarayeneh N et al. (2017) Integration of multi-omics data for integrative gene regulatory network inference. IJDMB 18(3), 223.
Zhang C et al. (2022) Deep latent space fusion for adaptive representation of heterogeneous multi-omics data. Briefings in Bioinformatics 23(2).
Zhang L et al. (2018) Deep Learning-Based Multi-Omics Data Integration Reveals Two Prognostic Subtypes in High-Risk Neuroblastoma. Front. Genet. 9.
Zhang L et al. (2021) AutoGGN: A gene graph network AutoML tool for multi-omics research. Artificial Intelligence in the Life Sciences 1, 100019.
Zhang X et al. (2019) Integrated Multi-omics Analysis Using Variational Autoencoders: Application to Pan-cancer Classification. 2019 IEEE International Conference on Bioinformatics and Biomedicine (BIBM).
Zhang X et al. (2021) OmiEmbed: A Unified Multi-Task Deep Learning Framework for Multi-Omics Data. Cancers 13(12), 3047.
Zhang X et al. (2021) Robust Prognostic Subtyping of Muscle-Invasive Bladder Cancer Revealed by Deep Learning-Based Multi-Omics Data Integration. Front. Oncol. 11.
Zhang Y et al. (2020) A Novel MKL Method for GBM Prognosis Prediction by Integrating Histopathological Image and Multi-Omics Data. IEEE J. Biomed. Health Inform. 24(1), 171-179.
Zhao L et al. (2021) DeepOmix: A scalable and interpretable multi-omics deep learning framework and application in cancer survival analysis. Computational and Structural Biotechnology Journal 19, 2719-2725.
Zhu C et al. (2022) DeepTpNet: A Deep Learning Approach to Identify Cancer Subtypes Based on Multi-omics Data. Proceedings of the 8th International Conference on Computing and Artificial Intelligence.

The 100 papers that were retrieved from the “ML AND integration” search

Abdullah M et al. (2019) The Classification of Skateboarding Trick Manoeuvres Through the Integration of IMU and Machine Learning. Lecture Notes in Mechanical Engineering, 67-74.

Abimbola O et al. (2020) Predicting Escherichia coli loads in cascading dams with machine learning: An integration of hydrometeorology, animal density and grazing pattern. Science of The Total Environment 722, 137894.
Acharjee A et al. (2016) Integration of metabolomics, lipidomics and clinical data using a machine learning method. BMC Bioinformatics 17(S15).
Akbari A et al. (2021) Drivers of economic and financial integration: A machine learning approach. Journal of Empirical Finance 61, 82-102.
Akolekar H et al. (2020) Integration of Machine Learning and Computational Fluid Dynamics to Develop Turbulence Models for Improved Turbine Wake Mixing Prediction. Volume 2C: Turbomachinery.
Alwadei S et al. (2022) Prediction of Solar Irradiance over the Arabian Peninsula: Satellite Data, Radiative Transfer Model, and Machine Learning Integration Approach. Applied Sciences 12(2), 717.
Andaryani S et al. (2021) Integration of hard and soft supervised machine learning for flood susceptibility mapping. Journal of Environmental Management 291, 112731.
Arbabshirani M et al. (2018) Advanced machine learning in action: identification of intracranial hemorrhage on computed tomography scans of the head with clinical workflow integration. npj Digital Med 1(1).
Biesbroek R et al. (2020) Machine learning for research on climate change adaptation policy integration: an exploratory UK case study. Reg Environ Change 20(3).
Boehm K et al. (2022) Multimodal data integration using machine learning improves risk stratification of high-grade serous ovarian cancer. Nat Cancer 3(6), 723-733.
Chaerkady R et al. (2021) Characterization of Citrullination Sites in Neutrophils and Mast Cells Activated by Ionomycin via Integration of Mass Spectrometry and Machine Learning. J. Proteome Res. 20(6), 3150-3164.
Chen H et al. (2021) A machine learning‐based survival prediction model of high grade glioma by integration of clinical and dose‐volume histogram parameters. Cancer Med 10(8), 2774-2786.
Chen J et al. (2021) Machine Learning–Based Digital Integration of Geotechnical and Ultrahigh–Frequency Geophysical Data for Offshore Site Characterizations. J. Geotech. Geoenviron. Eng. 147(12).
Cheng F et al. (2020) Slow pyrolysis as a platform for negative emissions technology: An integration of machine learning models, life cycle assessment, and economic analysis. Energy Conversion and Management 223, 113258.
Costache R et al. (2019) Identification of torrential valleys using GIS and a novel hybrid integration of artificial intelligence, machine learning and bivariate statistics. CATENA 183, 104179.
Costache R et al. (2020) Comparative assessment of the flash-flood potential within small mountain catchments using bivariate statistics and their novel hybrid integration with machine learning models. Science of The Total Environment 711, 134514.
Culos A et al. (2020) Integration of mechanistic immunological knowledge into a machine learning pipeline improves predictions. Nat Mach Intell 2(10), 619-628.
de Oliveira E et al. (2021) Integration of statistical inferences and machine learning algorithms for prediction of metritis cure in dairy cows. Journal of Dairy Science 104(12), 12887-12899.

Ding D et al. (2021) The first step towards intelligent wire arc additive manufacturing: An automatic bead modelling system using machine learning through industrial information integration. Journal of Industrial Information Integration 23, 100218.

Droutsas I et al. (2022) Integration of machine learning into process-based modelling to improve simulation of complex crop responses. in silico Plants 4(2).

Dullaghan C et al. (2017) Integration of machine learning techniques to evaluate dynamic customer segmentation analysis for mobile customers. arXiv preprint arXiv:1702.02215.

Fang Z et al. (2020) Integration of convolutional neural network and conventional machine learning classifiers for landslide susceptibility mapping. Computers & Geosciences 139, 104470.

Feng P et al. (2019) Machine learning-based integration of remotely-sensed drought factors can improve the estimation of agricultural drought in South-Eastern Australia. Agricultural Systems 173, 303-316.

Feng P et al. (2020) Machine learning-based integration of large-scale climate drivers can improve the forecast of seasonal rainfall probability in Australia. Environ. Res. Lett. 15(8), 084051.

Fillmore N et al. (2019) Interactive Machine Learning for Laboratory Data Integration. MedInfo, 264, 133-137.

Fu Y et al. (2020) Integration of an interpretable machine learning algorithm to identify early life risk factors of childhood obesity among preterm infants: a prospective birth cohort. BMC Med 18(1).

Gaikwad T et al. (2019) Vehicle Velocity Prediction and Energy Management Strategy Part 2: Integration of Machine Learning Vehicle Velocity Prediction with Optimal Energy Management to Improve Fuel Economy. SAE Technical Paper Series.

Gal A et al. (2018) Heterogeneous Data Integration by Learning to Rerank Schema Matches. 2018 IEEE International Conference on Data Mining (ICDM).

Ghezelbash R et al. (2019) Performance evaluation of RBF- and SVM-based machine learning algorithms for predictive mineral prospectivity modeling: integration of S-A multifractal model and mineralization controls. Earth Sci Inform 12(3), 277-293.

Gómez-Orellana A et al. (2021) Building Suitable Datasets for Soft Computing and Machine Learning Techniques from Meteorological Data Integration: A Case Study for Predicting Significant Wave Height and Energy Flux. Energies 14(2), 468.

Honorio J et al. (2015) Integration of PCA with a Novel Machine Learning Method for Reparameterization and Assisted History Matching Geologically Complex Reservoirs. Day 2 Tue, September 29, 2015.

Huang C et al. (2021) Real-time forecasting of suspended sediment concentrations reservoirs by the optimal integration of multiple machine learning techniques. Journal of Hydrology: Regional Studies 34, 100804.

Huang J et al. (2019) Application and comparison of several machine learning algorithms and their integration models in regression problems. Neural Comput & Applic 32(10), 5461-5469.

Hui G et al. (2021) Machine learning-based production forecast for shale gas in unconventional reservoirs via integration of geological and operational factors. Journal of Natural Gas Science and Engineering 94, 104045.

Iizuka K et al. (2020) Integration of Multi-Sensor Data to Estimate Plot-Level Stem Volume Using Machine Learning Algorithms–Case Study of Evergreen Conifer Planted Forests in Japan. Remote Sensing 12(10), 1649.
Jamthikar A et al. (2019) A low-cost machine learning-based cardiovascular/stroke risk assessment system: integration of conventional factors with image phenotypes. Cardiovasc. Diagn. Ther. 9(5), 420-430.
Jing W et al. (2015) Mapping Urban Areas with Integration of DMSP/OLS Nighttime Light and MODIS Data Using Machine Learning Techniques. Remote Sensing 7(9), 12419-12439.
Juárez-Orozco L et al. (2017) Quantitative myocardial perfusion evaluation with positron emission tomography and the risk of cardiovascular events in patients with coronary artery disease: a systematic review of prognostic studies. European Heart Journal - Cardiovascular Imaging 19(10), 1179-1187.
Kaczmarek I et al. (2022) A machine learning approach for integration of spatial development plans based on natural language processing. Sustainable Cities and Society 76, 103479.
Khan T et al. (2021) Effect of climate change on fruit by co-integration and machine learning. IJCCSM 13(2), 208-226.
Kloosterman A et al. (2020) Expansion of RiPP biosynthetic space through integration of pan-genomics and machine learning uncovers a novel class of lanthipeptides. PLoS Biol 18(12), e3001026.
Ko T et al. (2017) Machine learning-based anomaly detection via integration of manufacturing, inspection and after-sales service data. IMDS 117(5), 927-945.
Kropiwnicki E et al. (2021) Drugmonizome and Drugmonizome-ML: integration and abstraction of small molecule attributes for drug enrichment analysis and machine learning. Database 2021.
Leal L et al. (2019) Identification of disease-associated loci using machine learning for genotype and network data integration. Bioinformatics 35(24), 5182-5190.
León C et al. (2021) Studies of Shape Memory Graphene Nanostructures via Integration of Physics-based Modelling and Machine Learning. 9th edition of the International Conference on Computational Methods for Coupled Problems in Science and Engineering.
Li Y et al. (2019) Landslide Susceptibility Evaluation Using Hybrid Integration of Evidential Belief Function and Machine Learning Techniques. Water 12(1), 113.
Lin T et al. (2020) Covid-19 Chest Radiography Images Analysis Based on Integration of Image Preprocess, Guided Grad-CAM, Machine Learning and Risk Management. Proceedings of the 4th International Conference on Medical and Health Informatics.
Liu W et al. (2021) Multi-feature integration and machine learning for guided wave structural health monitoring: Application to switch rail foot. Structural Health Monitoring 20(4), 2013-2034.
Liu X et al. (2020) Mal-Prec: computational prediction of protein Malonylation sites via machine learning based feature integration. BMC Genomics 21(1).
Liu Z et al. (2022) Machine learning-based integration develops an immune-derived lncRNA signature for improving outcomes in colorectal cancer. Nat Commun 13(1).
Louca P et al. (2022) Machine learning integration of multimodal data identifies key features of blood pressure regulation. eBioMedicine 84, 104243.

Maj C et al. (2019) Integration of Machine Learning Methods to Dissect Genetically Imputed Transcriptomic Profiles in Alzheimer’s Disease. Front. Genet. 10.
Manap H et al. (2022) Data Integration for Lithological Mapping Using Machine Learning Algorithms. Earth Sci Inform 15(3), 1841-1859.
Mardani M et al. (2019) Integration of Machine Learning and Open Access Geospatial Data for Land Cover Mapping. Remote Sensing 11(16), 1907.
Masuda T et al. (2019) Machine-learning integration of CT histogram analysis to evaluate the composition of atherosclerotic plaques: Validation with IB-IVUS. Journal of Cardiovascular Computed Tomography 13(2), 163-169.
McIntosh C et al. (2021) Clinical integration of machine learning for curative-intent radiation treatment of patients with prostate cancer. Nat Med 27(6), 999-1005.
Mirzaei A et al. (2022) Healthcare data integration using machine learning: A case study evaluation with health information-seeking behavior databases. Research in Social and Administrative Pharmacy 18(12), 4144-4149.
Mishra C et al. (2021) Machine Learning Integration With Combustion Physics to Develop a Composite Predictive Model for Reactivity Controlled Compression Ignition Engine. Journal of Energy Resources Technology 144(4).
Mohammadi-Dehcheshmeh M et al. (2018) Unified Transcriptomic Signature of Arbuscular Mycorrhiza Colonization in Roots of Medicago truncatula by Integration of Machine Learning, Promoter Analysis, and Direct Merging Meta-Analysis. Front. Plant Sci. 9.
Mollalo A et al. (2020) Predicting the hotspots of age-adjusted mortality rates of lower respiratory infection across the continental United States: Integration of GIS, spatial statistics and machine learning algorithms. International Journal of Medical Informatics 142, 104248.
Mondal I et al. (2022) Core-log integration and application of machine learning technique for better reservoir characterisation of Eocene carbonates, Indian offshore. Energy Geoscience 3(1), 49-62.
Murali P et al. (2020) Integration of RNN with GARCH refined by whale optimization algorithm for yield forecasting: a hybrid machine learning approach. J Ambient Intell Human Comput.
Naimi S et al. (2021) Ground Observations and Environmental Covariates Integration for Mapping of Soil Salinity: A Machine Learning-Based Approach. Remote Sensing 13(23), 4825.
Nazemzadeh N et al. (2021) Integration of first-principle models and machine learning in a modeling framework: An application to flocculation. Chemical Engineering Science 245, 116864.
Nishi M et al. (2021) Prediction of well-being and insight into work-life integration among physicians using machine learning approach. PLoS ONE 16(7), e0254795.
Othman A et al. (2017) Integration of spectral, spatial and morphometric data into lithological mapping: A comparison of different Machine Learning Algorithms in the Kurdistan Region, NE Iraq. Journal of Asian Earth Sciences 146, 90-102.
Panahi B et al. (2019) Integration of Cross Species RNA-seq Meta-Analysis and Machine-Learning Models Identifies the Most Important Salt Stress–Responsive Pathways in Microalga Dunaliella. Front. Genet. 10.

Patidar R et al. (2021) An integration of geospatial and machine learning techniques for mapping groundwater potential: a case study of the Shipra river basin, India. Arab J Geosci 14(16).
Peng Z et al. (2020) A first look at the integration of machine learning models in complex autonomous driving systems: a case study on Apollo. Proceedings of the 28th ACM Joint Meeting on European Software Engineering Conference and Symposium on the Foundations of Software Engineering.
Pham B et al. (2017) Hybrid integration of Multilayer Perceptron Neural Networks and machine learning ensembles for landslide susceptibility assessment at Himalayan area (India) using GIS. CATENA 149, 52-63.
Pham T et al. (2018) Estimating aboveground biomass of a mangrove plantation on the Northern coast of Vietnam using machine learning techniques with an integration of ALOS-2 PALSAR-2 and Sentinel-2A data. International Journal of Remote Sensing 39(22), 7761-7788.
Pillay T et al. (2021) Integration of machine learning using hydroacoustic techniques and sediment sampling to refine substrate description in the Western Cape, South Africa. Marine Geology 440, 106599.
Race A et al. (2021) Deep Learning-Based Annotation Transfer between Molecular Imaging Modalities: An Automated Workflow for Multimodal Data Integration. Anal. Chem. 93(6), 3061-3071.
Rafiei M et al. (2016) A Novel Machine Learning Model for Estimation of Sale Prices of Real Estate Units. J. Constr. Eng. Manage. 142(2).
Riaz F et al. (2022) Identification of Secondary Breast Cancer in Vital Organs through the Integration of Machine Learning and Microarrays. Electronics 11(12), 1879.
Rodríguez‐Ortega A et al. (2021) Machine Learning‐Based Integration of Prognostic Magnetic Resonance Imaging Biomarkers for Myometrial Invasion Stratification in Endometrial Cancer. Magnetic Resonance Imaging 54(3), 987-995.
Sacco C et al. (2019) Machine learning based AFP inspection: A tool for characterization and integration (No. NF1676L-32137).
Saez Y et al. (2019) Integration in the European electricity market: A machine learning-based convergence analysis for the Central Western Europe region. Energy Policy 132, 549-566.
Shabanpour N et al. (2022) Integration of machine learning algorithms and GIS-based approaches to cutaneous leishmaniasis prevalence risk mapping. International Journal of Applied Earth Observation and Geoinformation 112, 102854.
Shamshiri R et al. (2020) Improving tropospheric corrections on large-scale Sentinel-1 interferograms using a machine learning approach for integration with GNSS-derived zenith total delay (ZTD). Remote Sensing of Environment 239, 111608.
Sharifi S et al. (2018) Integration of machine learning and meta-analysis identifies the transcriptomic bio-signature of mastitis disease in cattle. PloS one, 13(2), e0191227.
Showalter K et al. (2020) Machine learning integration of scleroderma histology and gene expression identifies fibroblast polarisation as a hallmark of clinical severity and improvement. Ann Rheum Dis 80(2), 228-237.

Singleton A et al. (2020) Mapping the geodemographics of digital inequality in Great Britain: An integration of machine learning into small area estimation. Computers, Environment and Urban Systems 82, 101486.

Sresakoolchai J et al. (2021) Integration of Building Information Modeling and Machine Learning for Railway Defect Localization. IEEE Access 9, 166039-166047.

Tamarappoo B et al. (2021) Machine learning integration of circulating and imaging biomarkers for explainable patient-specific prediction of cardiac events: A prospective study. Atherosclerosis 318, 76-82.

Tandon K et al. (2022) Integration of machine learning and particle filter approaches for forecasting soil moisture. Stoch Environ Res Risk Assess 36(12), 4235-4253.

Tao H et al. (2021) Integration of extreme gradient boosting feature selection approach with machine learning models: application of weather relative humidity prediction. Neural Comput & Applic 34(1), 515-533.

van Rosendael A et al. (2018) Maximization of the usage of coronary CTA derived plaque information using a machine learning based algorithm to improve risk stratification; insights from the CONFIRM registry. Journal of Cardiovascular Computed Tomography 12(3), 204-209.

Wadoux A et al. (2019) Multi-source data integration for soil mapping using deep learning. SOIL 5(1), 107-119.

Wang H et al. (2020) A New Approach for Unqualified Salted Sea Cucumber Identification: Integration of Image Texture and Machine Learning under the Pressure Contact. Journal of Sensors 2020, 1-13.

Wardeh M et al. (2020) Integration of shared-pathogen networks and machine learning reveals the key aspects of zoonoses and predicts mammalian reservoirs. Proc. R. Soc. B. 287(1920), 20192882.

Xie L et al. (2022) Wildfire Risk Assessment in Liangshan Prefecture, China Based on An Integration Machine Learning Algorithm. Remote Sensing 14(18), 4592.

Xu R (2020) Integration of NMR and conventional logs for vuggy facies classification in the arbuckle formation: a machine learning approach. *SPE Reservoir Evaluation & Engineering*, *23*(03), 917-929.

Xu X et al. (2019) Analysis of Thermophysical Properties of Deep Eutectic Solvents by Data Integration. J. Chem. Eng. Data 65(3), 1172-1179.

Yoo H et al. (2020) Deep learning-based evolutionary recommendation model for heterogeneous big data integration. KSII Transactions on Internet and Information Systems (TIIS), 14(9), 3730-3744.

Yousaf A et al. (2021) An Improved Residential Electricity Load Forecasting Using a Machine-Learning-Based Feature Selection Approach and a Proposed Integration Strategy. Sustainability 13(11), 6199.

Yousef M et al. (2021) miRcorrNet: machine learning-based integration of miRNA and mRNA expression profiles, combined with feature grouping and ranking. PeerJ 9, e11458.

Zhao H et al. (2019) Integration of machine learning approaches for accelerated discovery of transition-metal dichalcogenides as Hg0 sensing materials. *Applied Energy*, *254*, 113651.

Zhao L et al. (2021) Fluid and lithofacies prediction based on integration of well-log data and seismic inversion: A machine-learning approach. GEOPHYSICS 86(4), M151-M165.

Zou Y et al. (2019) Machine Learning-Based Integration of High-Resolution Wildfire Smoke Simulations and Observations for Regional Health Impact Assessment. IJERPH 16(12), 2137.
